# Ileal mucosa-associated microbiota overgrowth associated with pathogenesis of primary biliary cholangitis

**DOI:** 10.1038/s41598-021-99314-9

**Published:** 2021-10-05

**Authors:** Shogo Kitahata, Yasunori Yamamoto, Osamu Yoshida, Yoshio Tokumoto, Tomoe Kawamura, Shinya Furukawa, Teru Kumagi, Masashi Hirooka, Eiji Takeshita, Masanori Abe, Yoshiou Ikeda, Yoichi Hiasa

**Affiliations:** 1 Department of Gastroenterology and Metabology, Ehime University Graduate School of Medicine, Shitsukawa, Toon, Ehime, 791-0295 Japan; 2 Endoscopy Center, Ehime University Graduate School of Medicine, Ehime, Japan; 3 Department of Community Medicine, Ehime University Graduate School of Medicine, Ehime, Japan; 4 Health Services Center, Ehime University, Ehime, Japan; 5 Ehime University Hospital Postgraduate Medical Education Center, Ehime, Japan; 6 Department of Inflammatory Bowel Diseases and Therapeutics, Ehime University Graduate School of Medicine, Ehime, Japan

**Keywords:** Primary biliary cirrhosis, Dysbiosis

## Abstract

The small intestinal mucosa-associated microbiota (MAM) can potentially impact the etiology of primary biliary cholangitis (PBC). Herein, we investigate the MAM profile to determine its association with liver pathology in patients with PBC. Thirty-four patients with PBC and 21 healthy controls who underwent colonoscopy at our hospital were enrolled in our study. We performed 16S ribosomal RNA gene sequencing of MAM samples obtained from the mucosa of the terminal ileum and examined the relationship between the abundance of ileal MAM and chronic nonsuppurative destructive cholangitis using liver specimens from patients with PBC. There was a significant reduction in microbial diversity within individuals with PBC (*P* = 0.039). Dysbiosis of ileal MAM was observed in patients with PBC, with a characteristic overgrowth of *Sphingomonadaceae* and *Pseudomonas*. Multivariate analysis showed that the overgrowth of *Sphingomonadaceae* and *Pseudomonas* is an independent association factor for PBC (*P* = 0.0429, *P* = 0.026). Moreover, the abundance of *Sphingomonadaceae* was associated with chronic nonsuppurative destructive cholangitis in PBC (*P* = 0.00981). The overgrowth of *Sphingomonadaceae* and *Pseudomonas* in ileal MAM was found in patients with PBC. *Sphingomonadaceae* may be associated with the pathological development of PBC.

## Introduction

Primary biliary cholangitis (PBC) is a chronic liver disease resulting from progressive, immune-mediated destruction of the small interlobular bile ducts. It leads to progressive intrahepatic cholestasis and eventually fibrosis and cirrhosis of the liver^1,2^. The etiology of PBC is attributed to a combination of genetic predispositions and environmental triggers such as the gut microbiota^3^. A characteristic of this disease is the presence of antimitochondrial antibodies (AMA), which are autoantibodies against mitochondrial pyruvate dehydrogenase complex E2 (PDC-E2)^4,5^. Previous studies have reported that *Novosphingobium aromaticivorans*, a bacterium belonging to the family of *Sphingomonadaceae*, produced a homologous enzyme against PDC-E2. Moreover, mice infected with *N. aromaticivorans* show antibodies against microbial PDC-E2 and its mitochondrial counterpart, as well as chronic T cell-mediated autoimmunity against small bile ducts^9^. Fecal microbiota profiles of patients with PBC have a significant reduction in microbial diversity compared with healthy controls (HCs), suggesting gut microbiota as a potential therapeutic target and diagnostic biomarker for PBC^10,11^. However, overgrowth of any particular bacterium, including *Sphingomonadaceae*, has not been identified in the fecal microbiome of patients with PBC. Therefore, the infection site of the specific bacteria involved in the pathogenesis and progression of PBC remains unknown.

The gut microbiota comprises luminal microbiota (LM), fecal microbiota, and mucosa-associated microbiota (MAM) present in the intestinal mucosa. MAM affects epithelial and mucosal functions more remarkably than LM, and hence, it is potentially more involved in PBC pathophysiology than LM^12^. However, the MAM profile in patients with PBC is unknown. The ileal MAM could be associated with the synthesis of bile acids that are involved in PBC pathogenesis^13,14^. Most bile acids return to the liver via the enterohepatic circulation at the terminal ileum^15^. Thus, in cholestatic disease, the profile of ileal MAM is an important factor in the pathophysiology of this disease. Recently, a brushing method was proposed to evaluate MAM^16^. In the present study, we aimed to investigate the ileal MAM profile and identify its association with liver pathology in patients with PBC.

## Results

### Clinical characteristics of the subjects

The clinical characteristics of patients with PBC and HCs are summarized in Table 1. Although the number of women in the PBC group was significantly greater than that in the HC group, their mean age and body mass indices were comparable. The levels of γ-glutamyl transpeptidase, alkaline phosphatase (ALP), total bile acid, and immunoglobulin M were higher in patients with PBC than in those of the HCs. High levels of total bilirubin in the sera of HCs were observed, although within the standard range.

**Table 1:** Baseline characteristics of 34 patients with PBC and 21 healthy controls.

	PBC (n = 34)	Healthy control (n = 21)	*P* value
Age, years*	66 (58–72)	69 (55–73)	0.795
Gender, male/female	2/32	9/12	0.001
BMI, kg/m^2^*	22.4 (20.1–24.9)	22.8 (20.8–24.9)	0.822
ALT, U/L*	18 (14–22.8)	17 (15–22)	0.815
AST, U/L*	26.5 (22–30.8)	22 (19–26)	0.081
GGT, U/L*	34.5 (21–63.8)	19 (17–35)	0.01
ALP, U/L*	96.2 (78–115)	79 (63–86)	0.003
T-Bil, mg/dL*	0.6 (0.4–0.8)	0.8 (0.7–1.0)	0.016
PT, %*	105 (95–118)	103 (94.8–113)	0.457
Total bile acid, μmol/L*	11.9 (8–18.7)	4.1 (2.8–6.9)	0.0001
IgM, mg/dL*	137.5 (96.7–192)	103 (52–141.5)	0.044
IgG, mg/dL*	1315 (1162–1526)	1350 (1245–1470)	0.67
AMA, n (+ %)	26/8 (76.5%)		
UDCA, n (+ %)	28/6 (82.4%)		
UDCA responders**/non responders/not available	20/1/7		
Clinical cirrhosis, n (+ %)	0/34 (0%)		

Wilcoxon rank-sum test was used to compare age, BMI, and blood test results between PBC and healthy controls; fisher’s exact test was used to compare gender distribution between PBC and controls.

PBC, primary biliary cholangitis; BMI, body mass index; ALT, alanine aminotransferase; AST, aspartate aminotransferase; GGT, γ-glutamyltranspeptidase; ALP, alkaline phosphatase; T-Bil, total bilirubin; PT, prothrombin time; IgM, immunoglobulin M; IgG, immunoglobulin G; AMA, antimitochondria antibody; UDCA, ursodeoxycholic acid.

*Median (interquartile range).

**UDCA responders were evaluated based on the Paris-II criteria.

### Dysbiosis in the MAM of patients with PBC

**Figure 1:**
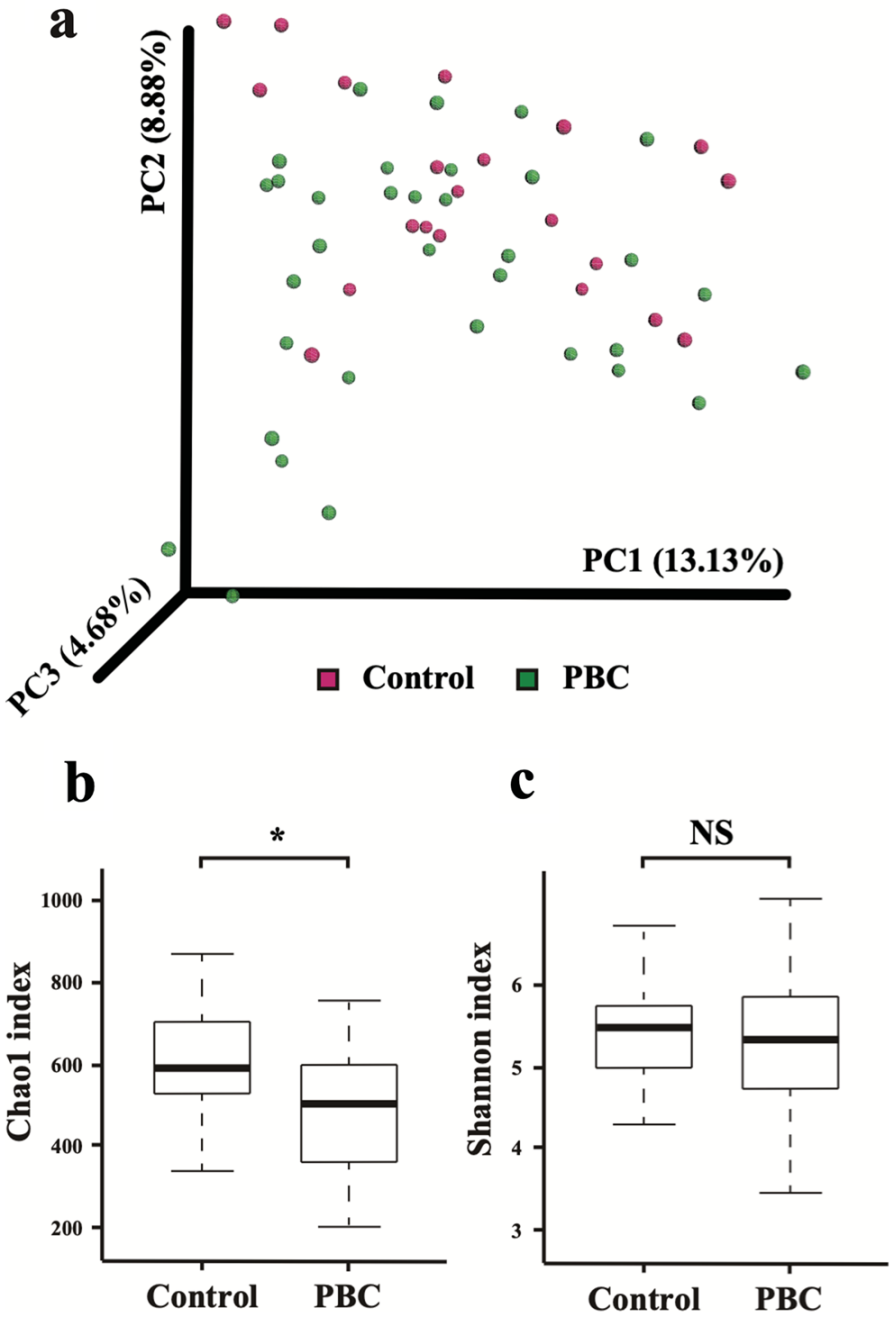
Comparison of MAM diversity between patients with PBC and HCs. (**a**) Principal coordinate analysis of unweighted UniFrac analysis using Emperor (0.9.60, http://biocore.github.io/emperor/build/html/scripts/make_emperor.html) showed a somewhat weak clustering of the MAM between the patients with PBC and HCs (pseudo-F: 1.44, *P* = 0.054, PERMANOVA). (**b**) Changes in the mucosa-associated microbiota diversities in patients with PBC (*n* = 34) compared with HCs (*n* = 21). An α-diversity, illustrated by microbial richness (Chao-1 index), was reduced in the PBC group (*P* = 0.039, Wilcoxon rank-sum test). *P < 0.05. (**c**) No difference in index (Shannon) of diversity. MAM, mucosa-associated microbiota; PBC, primary biliary cholangitis; HC, healthy control; PERMANOVA, permutational analysis of variance.

An unweighted, UniFrac-based, principal coordinate analysis showed relatively weak clustering of MAM between patients with PBC and HCs (PERMANOVA, pseudo-F: 1.44, *P* = 0.054, Fig. 1a).

Gut microbiota richness, determined by the Chao-1 index, was lower in patients with PBC than in HCs (*P* = 0.039, Wilcoxon rank-sum test; Fig. 1b). However, the Shannon index, which measures both richness and evenness, was not significantly different among the two groups (*P* > 0.05, Fig. 1c).

### Characterization of MAM present in patients with PBC and HCs

**Figure 2:**
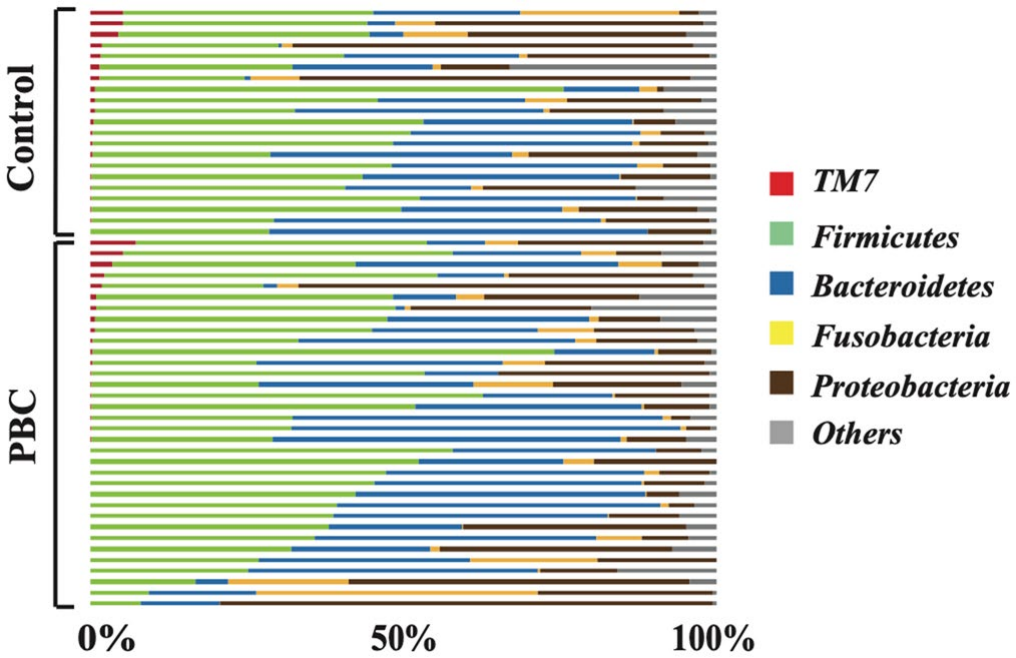
Differences in characterized MAM between patients with PBC and HCs at the phylum level. Bacterial composition at phylum level. The relative amount of bacterial composition at the phylum level is shown in a bar graph for each sample obtained from the PBC and HC groups. MAM, mucosa-associated microbiota; PBC, primary biliary cholangitis; HC, healthy control.

To estimate microbiota diversity in PBC patient samples, we assessed the relative abundance of taxa in both groups. Figure 2 shows the relative bacterial abundance at the phylum level in the samples from both groups. The most dominant phylum was *Firmicutes* with an average relative abundance of 40.5% and 40.3% in the PBC and HC groups, respectively.

**Figure 3:**
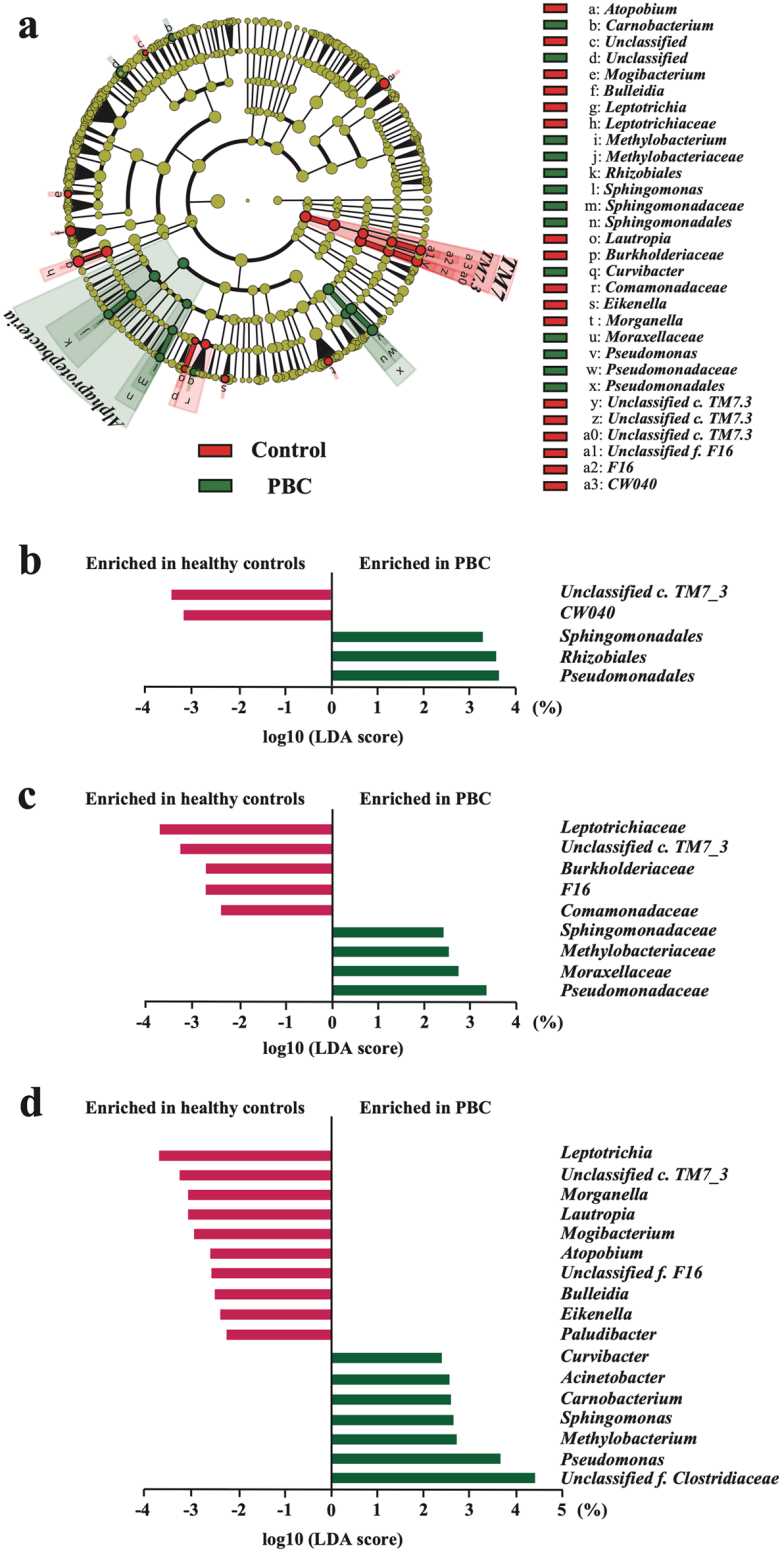
Changes in gut bacterial taxonomic abundance in patients with PBC (*n* = 34) compared with HCs (*n* = 21). (**a**) Bacterial taxa identified to be rich based on the differences between the groups analyzed by LEfSe (LEfSe v1.0, https//huttenhower.sph.harvard.edu/lefse/) (Logarithmic LDA score > 2.0). Bacterial taxa of the patients with PBC were compared with those of HCs at various levels. Bacterial taxa with high numbers of bacteria in the PBC group are shown in green, and bacterial taxa with high numbers of bacteria in the HC group are shown in red. In addition, the bacterial taxa showing insignificant differences in their abundance between the two groups are shown in yellow. LEfSe identified *Alphaproteobacteria* as a differentially abundant taxon in the PBC group versus the HC group. (**b**) Order-level relative abundance of bacteria in the PBC group compared with that in the HC group. (**c**) Family-level relative abundance of bacteria in the PBC group compared with that in the HC group. (**d**) Genus-level relative abundance of bacteria in the PBC group compared with that in the HC group. Values are expressed as the median of the interquartile range. PBC, primary biliary cholangitis; HC, healthy control; LEfSe, linear discriminant analysis effect size; LDA, linear discriminant analysis.

The differentially abundant taxa between the control and PBC groups were identified using the linear discriminant analysis (LDA) effect size (LEfSe) (minimum LDA score: 2.0). We identified 34 taxa, including 1 phylum, 2 classes, 5 orders, 9 families, and 17 genera, all of which were significantly abundant between the groups. At the phylum level, *TM7* was lower in the PBC group than in the HC group (Fig. 3a). At the class level, *Alphaproteobacteria* were more abundant in the PBC group than in the HC group, whereas *TM7_3* was significantly decreased in the PBC group (Fig. 3a). At the order level, *Sphingomonadales* and *Rhizobiales*, of the class *Alphaproteobacteria* and *Pseudomonadales*, respectively, were more abundant in the PBC group than in the HC group. *CW040* and a species belonging to an unknown order that belongs to the *TM7_3* class were considerably decreased in the PBC group (Fig. 3b). At the family level, four bacterial taxa including *Sphingomonadaceae, Pseudomonadaceae*, *Methylobacteriaceae*, and *Moraxellaceae* were more abundant in the PBC group than in the HC group, whereas *Leptotrichiaceae*, *Burkholderiaceae*, *Comamonadaceae*, *F16*, and an unknown family belonging to the class *TM7_3* were considerably decreased in the PBC group (Fig. 3c). The abundance of 17 bacterial genera differed between the groups (Fig. 3d, Supplementary Table S1). Ten genera (*Leptotrichia, Morganella*, *Lautropia*, *Mogibacterium*, *Atopobium*, *Bulleidia*, *Eikenella*, *Paludibacter*, an unknown genus belonging to the class *TM7_3*, and *F16,g* (an unknown genus of the family *F16*) were substantially abundant in HCs (Fig. 3d, red bar, Supplementary Table S1). Conversely, seven genera (*Sphingomonas*, *Pseudomonas*, *Methylobacterium*, *Carnobacterium*, *Acinetobacter*, *Curvibacter*, and the unknown genus *Clostridiaceae* belonging to the family *Clostridiaceae*) were substantially abundant in the PBC group (Fig. 3d, green bar, Supplementary Table S1). Notably, *Sphingomonas* and *Pseudomonas* were associated with PBC from the order to the genus levels. Of these, we further focused on *Sphingomonadaceae* and *Pseudomonas*, which have been previously reported to be related to PBC^9^.

### Relationship between specific bacteria and treatment-naïve PBC

To rule out the effects of therapeutic agents, we evaluated the relative abundance of *Sphingomonadaceae* and *Pseudomonas* in treatment-naïve patients with PBC or patients receiving UDCA, the main treatment for PBC. No significant difference was observed when the abundance of *Sphingomonadaceae* and *Pseudomonas* was compared between the UDCA-naïve PBC (n = 6) and UDCA-treated (n = 28) groups (Supplementary Fig. S1, Supplementary Table S2).

## Analysis of association factors contributing to PBC using microbiota signature

To explore whether the gut microbiome can help determine the PBC status, we determined the abundance of *Sphingomonadaceae* and *Pseudomonas* in the PBC groups by receiver operating characteristic analysis. The AUC values obtained were 0.745 (95% CI 0.63–0.87, Supplementary Fig. S2a) and 0.735 (95% CI 0.60–0.87, Supplementary Fig. S2b), respectively. The cut-off values were 0.0000179 and 0.0000684 for *Sphingomonadaceae* and *Pseudomonas*, respectively. Based on this threshold, patients with PBC were divided into four subgroups, i.e., *Sphingomonadaceae* rich and deficient, and *Pseudomonas* rich and deficient. Since PBC frequently occurs in middle-aged women, univariable analysis of these bacteria was performed considering age and sex as the evaluation parameters. Factors (*Sphingomonadaceae* rich, *Pseudomonas* rich, and sex) identified using univariable analysis were subject to multivariable analysis. Among *Sphingomonadaceae*, *Pseudomonas*, and sex, which were significant in univariate analysis, all items remained significant after multivariable analysis (*P* = 0.0429, *P* = 0.026, *P* = 0.0138, Table 2).

**Table 2:** Analysis of association factors contributing to PBC.

	PBC rate (%)	Crude OR (95% CI)	Adjusted OR* (95% CI)
*Sphingomonadaceae* deficient**	13/31 (41.9)	1.0	1.0
*Sphingomonadaceae* rich**	21/24 (87.5)	9.69 (2.38–39.5)	5.06 (1.05–24.3)
*p* value		0.00153	0.0429
*Pseudomonas* deficient***	6/19 (31.6)	1.0	1.0
*Pseudomonas* rich***	28/36 (77.8)	7.58 (2.18–16.4)	6.7 (1.26–35.8)
*p* value		0.00144	0.026
male	2/11 (18.2)	1.0	1.0
female	32/44 (72.7)	12 (2.26–63.7)	16 (1.76–146)
*p* value		0.00353	0.0138
age		1.0 (0.95–1.05)	1.06 (0.98–1.15)
*p* value		0.96	0.12
ALP ****≦ 113	25/45 (55.5)	1.0	
ALP **** > 113	9/10 (90.0)	7.2 (0.84–61.7)	
*p* value		0.0716	

*Logistic regression test was adjusted for covariates including gender and age.

**The cutoff values were 0.0000179.

***The cutoff values were 0.0000684.

****The cutoff value of ALP was set as the upper limit of normal, which is the diagnostic criterion for PBC.

PBC, primary biliary cholangitis; OR, odds ratio; CI, confidence interval; ALP, alkaline phosphatase.

### Relationship between specific bacteria and chronic non-suppurative destructive cholangitis (CNSDC)

**Figure 4:**
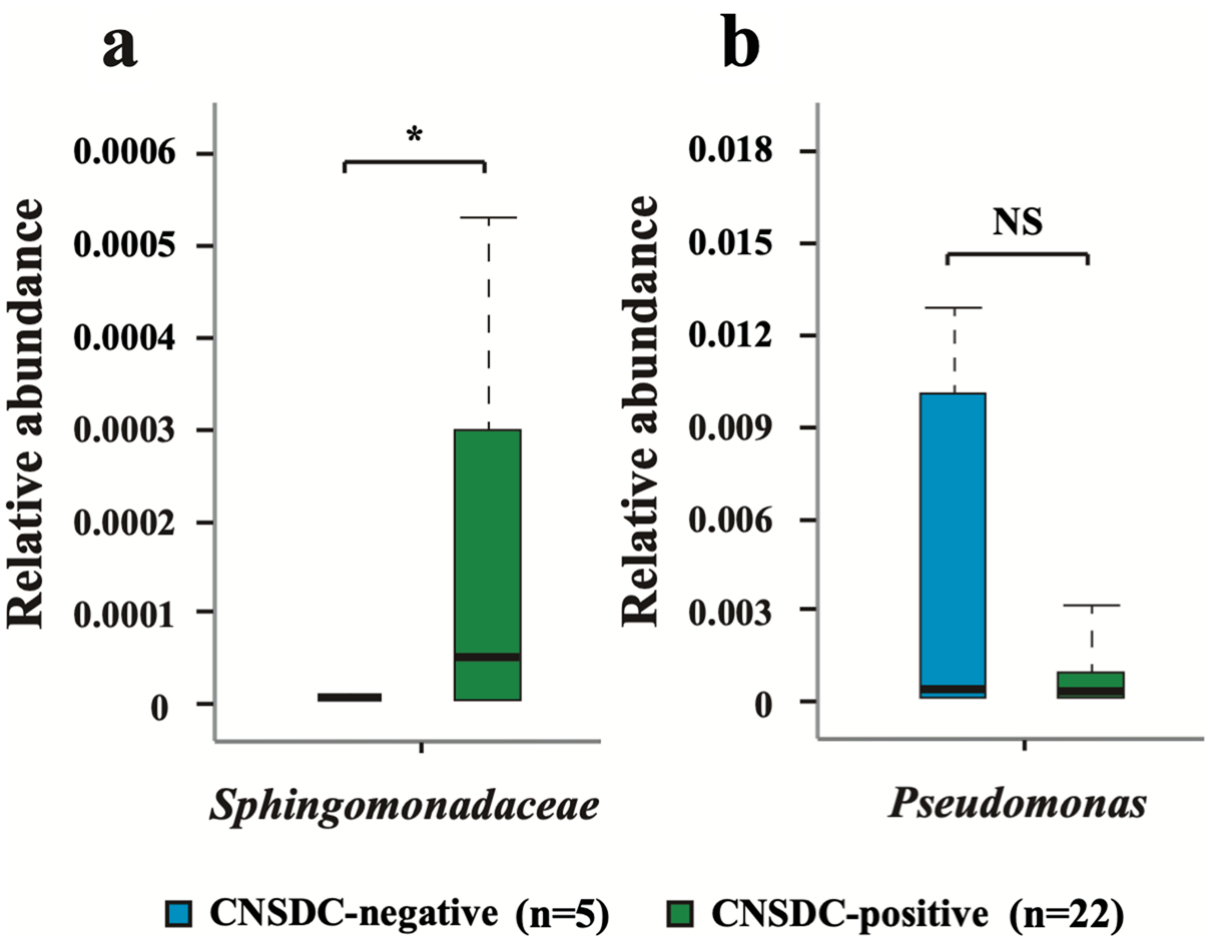
Relative abundance of (**a**) *Sphingomonadaceae* and (**b**) *Pseudomonas* in the CNSDC-positive PBC group compared with that in the CNSDC-negative PBC group. The values are expressed as the median of the interquartile range. *P < 0.05 compared with the CNSDC-negative PBC group. CNSDC, chronic non-suppurative destructive cholangitis; PBC, primary biliary cholangitis.

We evaluated the relative abundance of *Sphingomonadaceae* and *Pseudomonas* in 27 patients who had undergone liver biopsy to investigate whether these bacteria are associated with the pathology of PBC. A comparison of the abundance of *Sphingomonadaceae* in CNSDC-positive PBC (n = 22) and CNSDC-negative PBC (n = 5) groups showed that *Sphingomonadaceae* were more abundant in the CNSDC-positive group than in the CNSDC-negative group (*P* = 0.0194, Fig. 4a). Similar results were obtained with the *Sphingomonadaceae*-rich group based on the cut-off value obtained by ROC (*P* = 0.00981, Table 3). On the other hand, there was no difference in the abundance of *Pseudomonas* between the CNSDC-positive group and CNSDC-negative group (Fig. 4b).

**Table 3:** Relationship between the mucosa-associated microbiota and CNSDC.

	CNSDC positive (n = 22)	CNSDC negative (n = 5)	*P* value*
*Sphingomonadaceae* Rich/Deficient**	15/7	0/5	0.00981
*Pseudomonas* Rich/Deficient***	18/4	4/1	1

CNSDC, chronic non-suppurative destructive cholangitis.

*Comparisons using Fisher’s exact test.

**The cutoff values were 0.0000179.

***The cutoff values were 0.0000684.

## Discussion

This study was designed to evaluate the ileal MAM profile and identify its association with liver pathology in patients with PBC. We found dysbiosis in the ileal MAM of patients with PBC. Furthermore, the abundance of *Sphingomonadaceae* and *Pseudomonas* in the MAM of patients with PBC was significantly increased compared with that in HCs and showed an independent association for PBC. In particular, the amount of *Sphingomonadaceae* was associated with the CNSDC in the liver biopsy samples of patients with PBC. Previous studies have reported a significant reduction in intra-individual microbial diversity in the fecal samples of patients with PBC^10,11^. This finding was consistent with the results of the present study. However, overgrowth of any particular bacteria involved in the pathogenesis and progression of PBC has not been identified in the fecal microbiota of patients with PBC. To the best of our knowledge, this study is the first to identify the overgrowth of *Sphingomonadaceae* and *Pseudomonas* in the MAM of patients with PBC.

The bacteria that belong to the division/phylum *Proteobacteria* and class *Alphaproteobacteria* have been remarkably associated with autoimmunity^17^. The cell wall of *Sphingomonadaceae* spp. and those belonging to the *Alphaproteobacteria* class is unusual as it contains glycosphingolipids (GSLs) instead of lipopolysaccharides^18,19^. The unique cell wall of these bacteria activates distinct innate immune pathways that may trigger the development of liver-specific pathological conditions^17^. Mice infected with *N. aromaticivorans*, belonging to the genus *Sphingomonadaceae*, developed antibodies against PDC-E2 and had liver histology similar to patients with PBC^9^. The model was postulated because natural killer T (NKT) cells specifically recognized the GSLs of various bacterial strains belonging to the genus *Sphingomonadaceae* through their semi-invariant CD1d-restricted T-cell receptor^9^. *Sphingomonadaceae* GSLs, which have a similar structure to α-GalCer, bind to mouse CD1d and stimulate Vα14iNKT cells independent of antigen-presenting cell activation and interleukin-12^20,21^. NKT cells accumulate in the liver^22^, and the proportion of NKT cells in the liver has been reported to be significantly higher in patients with PBC than in healthy individuals^23^. Additionally, CD1d is focally expressed on small bile duct epithelial cells in patients with PBC but not in healthy individuals^24^. Previous studies performed using fecal samples of patients with PBC reported no significant differences in the abundance of *Sphingomonadaceae*^10^. The MAM of the small intestine is different from the LM and is considered to be more deeply involved in the pathophysiology of PBC^16^. Our results showed that patients with PBC have significantly more *Sphingomonadaceae* than HCs. Additionally, the dominance of *Sphingomonadaceae* detected with receiver operating characteristic curve and multivariate analysis suggested *Sphingomonadaceae* as one of the factors involved in PBC. Our findings point towards the terminal ileum as the site of infection.

*Pseudomonas* primarily causes urinary tract infection, acts as an environmental risk factor for PBC, and causes the production of PBC autoantibodies^25–27^. Previous studies on patients with PBC showed extensive cross-reactivity between the dominant B- and T-cell epitopes of human PDC-E2 and microbial mimics, suggesting the role of microbial infection in the induction of antimitochondrial antibodies through the mechanism of molecular mimicry^28^. *Pseudomonas aeruginosa*, *N. aromaticivorans*, and *Escherichia coli* contain proteins that are homologous with PDC-E2^8,9^. *Pseudomonas* species cross-reacting with PDC-E2-reactive CD4^+^ and CD8^+^ T cells have been identified and show potential agonistic effects^25,26^. A previous study identified *Pseudomonas* species in the liver tissue of patients with PBC^29^. *Pseudomonas* may therefore have a significant effect on immunological tolerance and autoimmunity in patients with PBC. The dominance of *Pseudomonas* analyzed through receiver operating characteristic curve and multivariate analysis suggested that *Pseudomonas* is one of the factors involved in PBC etiology similar to *Sphingomonadaceae*.

CNSDC, one of the diagnostic criteria for PBC, is a typical early hepatopathological finding of PBC. Previous studies have reported the production of bacterially induced PBC-like CNSDC in mice^30^, but there are no reports of an association between CNSDC and specific bacteria in humans. Our results illustrate the association between CNSDC and *Sphingomonadaceae*, which stimulates NKT cells, although the number of cases is small. Bile duct epithelial staining of CD1d is a common feature in early-stage PBC but is rarely present in late-stage PBC^24^. Innate immunity triggered by *Sphingomonadaceae* may be a contributing factor for the pathophysiology of early PBC.

Our study has some limitations. First, we did not investigate cirrhosis and included limited cases of UDCA non-responders, hence, whether *Sphingomonadaceae* and *Pseudomonas* are involved in the onset and pathological progression and treatment responsiveness remains to be determined. Therefore, investigating an independent cohort comprising early- and late-stage PBC is warranted to verify the value of MAM in distinguishing between PBC and HC. Second, this was a cross-sectional study, and therefore, we cannot conclude that there is a causal relationship between MAM and PBC. Evaluation of the effect of treatment against *Sphingomonadaceae* and *Pseudomonas* to inhibit the development of PBC should be assessed in vivo. Third, the time of liver biopsy was different from that of bacterial collection. Although evaluation at the same time is optimal, the different time of collection has a small effect because the gut microbiota does not change unless there are antibiotics present or major events have occurred. Fourth, the number of cases included was insufficient to exclude the effects of UDCA. In addition, other cholestasis diseases, such as primary sclerosing cholangitis (PSC), were not evaluated. Changes in MAM observed in patients with PBC might have been attributed to cholestasis. The main strength of this study is that it is the first to identify the overgrowth of specific bacteria in ileal MAM of PBC, and these bacteria are potentially associated in the pathogenesis of PBC.

In conclusion, we revealed dysbiosis in the MAM profile of the small intestine in patients with PBC and characteristic bacterial overgrowth that has been reported to be associated with PBC. *Sphingomonadaceae* may be associated with the pathological development of PBC. Our results contribute to the development of effective antibiotic treatment strategies for PBC.

## Methods

### Subjects

A total of 41 patients with PBC and 24 HCs underwent colonoscopy for colorectal cancer screening at the Ehime University Hospital (Ehime, Japan) between March 2018 and January 2020. The selection criteria for the subjects were as follows: [a] Biochemical evidence of cholestasis (increase in the serum ALP level), [b] AMA positivity, and [c] histological evidence of the destruction of interlobular bile ducts and nonsuppurative destructive cholangitis per liver biopsy. Patients who fulfilled at least two of the three criteria were included in this study^31^. Liver biopsy was performed to determine disease activity, staging, and prognosis for all recruited PBC patients consented to liver biopsy at our hospital. Clinical cirrhosis was identified by either histological or imaging findings showing cirrhosis or portal hypertension, or if there were symptoms associated with cirrhosis. The exclusion criteria were as follows: viral hepatitis, autoimmune hepatitis, biliary tract system and pancreatic disease, inflammatory bowel disease, past surgical history of gastroenterological diseases, history of liver transplantation, and history of immunosuppressants use. Healthy subjects with no liver, gastrointestinal, or autoimmune diseases were recruited in this study. No subjects had received antibiotics or immunosuppressants for at least 2 months prior to enrolment. The 16S rRNA sequence analysis was conducted on 34 patients with PBC and 21 HCs (Supplementary Fig. S3). The groups were further subdivided based on whether patients received ursodeoxycholic acid (UDCA) treatment. In addition, we analyzed a subgroup of patients who had undergone liver biopsy. All the subjects provided written informed consent and the study protocol was developed in accordance with the ethical guidelines of the 1964 Declaration of Helsinki and later versions. All procedures followed were in accordance with the ethical standards of the responsible committee on human experimentation (institutional and national). This study was approved by the ethics committee of Ehime University of Medical Science (approval no. 1610012). All the patients were managed at the Division of Gastroenterology of Ehime University Hospital. The study was registered at the University Hospital Medical Information Network Center (UMIN 000040177). All authors had access to the study data and reviewed and approved the final manuscript.

### Sample collection and DNA extraction for MAM study

Samples from the terminal ileum were collected by gently brushing the mucosal surfaces using RX cytology brushes (Boston Scientific, Marlborough, MA, USA) according to a previously reported technique^16^ and frozen at − 80 °C. DNA extraction was performed using the QIAamp UCP Pathogen Mini Kit (Qiagen, Hilden, Germany).

### 16S ribosomal RNA (rRNA) gene sequencing

Using the MiSeq system (Illumina, San Diego, CA, USA), 16S rRNA gene sequencing was performed as described previously^32^. The V3–V4 region of the bacterial 16S ribosomal RNA gene was amplified using the KAPA HiFi HotStart PCR kit (Kapa Biosystems, Woburn, MA, USA) and barcode-indexed primers 341F (5′-CCTACGGGNGGCWGCAG-3′) and 806R (5′-GACTACHVGGGTATCTAATCC-3′). The amplicons were purified using AMpureXP (Beckman Coulter, Tokyo, Japan) and quantified using Qubit (Thermo Fisher Scientific, Waltham, MA, USA). The purified amplicons were pooled in equimolar concentrations (1.20 ng/μL) and sequenced.

### Data analysis

The 16S rRNA sequence analysis was performed using the QIIME suite of software tools (v1.9.1)^33^. Operational taxonomic units were picked from filtered sequence reads (Phred score ≥ Q33) by a closed-reference operational taxonomic unit-picking method on the basis of 97% identity with the Greengenes database (v13.5)^34^. Chimeric sequences were trimmed using the ChimeraSlayer method^35^. Chao-1 diversity indices were used to compare the diversity of gut microbiota profiles between patients with PBC and HCs. For comparing beta diversity, weighted and unweighted UniFrac distances were calculated^36^. For multivariate analysis of variance, permutational analysis of variance (PERMANOVA) was used with 999 permutations.

### Statistical analysis

All statistical analyses were performed using R package (V.3.5.0). For comparison of nominal variables, fisher's exact test was used, and continuous variables were executed using the paired Wilcoxon rank-sum test. Taxonomic differences between groups were assessed via LEfSe^37^. Using the LEfSe algorithm, bacterial taxa that were differentially abundant in the pairwise analysis for groups were first identified and tested using the Kruskal Wallis test with adjustments for multiple comparisons (*P* < 0.05). The identified features were then subjected to the LDA model with a threshold logarithmic LDA score set at 2.0 and ranked. Taxonomic levels with LEfSe values higher than 2 at *P* < 0.05 were considered statistically significant. To evaluate the discriminatory ability of the gut microbiota of patients with PBC, operating characteristic curves (receiving operational curve [ROC]) were constructed and the area under the curve (AUC) was calculated; the cutoff value was determined using the Youden index. Logistic regression tests were used to regress the relative abundance of taxon against age and gender.

## Supplementary Information

Supplementary Figure S1.

Supplementary Figure S2.

Supplementary Figure S3.

Supplementary Table S1.

Supplementary Table S2.

## Data Availability

The data that support the findings of this study are available from the corresponding author, YH, upon reasonable request.

## References

[1] Lindor KD (2009). Primary biliary cirrhosis. Hepatology.

[2] Kumagi T, Heathcote EJ (2008). Primary biliary cirrhosis. Orphanet. J. Rare Dis..

[3] Selmi C (2004). Primary biliary cirrhosis in monozygotic and dizygotic twins: Genetics, epigenetics, and environment. Gastroenterology.

[4] Oertelt S (2007). A sensitive bead assay for antimitochondrial antibodies: Chipping away at AMA-negative primary biliary cirrhosis. Hepatology.

[5] Moteki S (1996). Epitope mapping and reactivity of autoantibodies to the E2 component of 2-oxoglutarate dehydrogenase complex in primary biliary cirrhosis using recombinant 2-oxoglutarate dehydrogenase complex. Hepatology.

[6] Padgett KA (2005). Phylogenetic and immunological definition of four lipoylated proteins from *Novosphingobium aromaticivorans*, implications for primary biliary cirrhosis. J. Autoimmun..

[7] Kaplan MM (2004). Novosphingobium aromaticivorans: A potential initiator of primary biliary cirrhosis. Am. J. Gastroenterol..

[8] Selmi C (2003). Patients with primary biliary cirrhosis react against a ubiquitous xenobiotic-metabolizing bacterium. Hepatology.

[9] Mattner J (2008). Liver autoimmunity triggered by microbial activation of natural killer T cells. Cell Host Microbe..

[10] Tang R (2018). Gut microbial profile is altered in primary biliary cholangitis and partially restored after UDCA therapy. Gut.

[11] Furukawa M (2020). Gut dysbiosis associated with clinical prognosis of patients with primary biliary cholangitis. Hepatol. Res..

[12] Ringel Y, Maharshak N, Ringel-Kulka T, Wolber EA, Sartor RB, Caroll IM (2015). High throughput sequencing reveals distinct microbial populations within the mucosal and luminal niches in healthy individuals. Gut Microbes..

[13] Li Y, Tang R, Leung PSC, Gerschwin ME, Ma X (2017). Bile acids and intestinal microbiota in autoimmune cholestatic liver diseases. Autoimmun. Rev..

[14] Chen W (2020). Comprehensive analysis of serum and fecal bile acid profiles and interaction with gut microbiota in primary biliary cholangitis. Clin. Rev. Allergy Immunol..

[15] Hamer HM, De Preter V, Windey K, Verbeke K (2012). Functional analysis of colonic bacterial metabolism: relevant to health?. Am. J. Physiol. Gastrointest. Liver Physiol..

[16] Nishino K (2018). Analysis of endoscopic brush samples identified mucosa-associated dysbiosis in inflammatory bowel disease. J. Gastroenterol..

[17] Mohammed JP, Mattner J (2009). Autoimmune disease triggered by infection with alphaproteobacteria. Expert Rev. Clin. Immunol..

[18] Kawahara K, Kuraishi H, Zähringer U (1999). Chemical structure and function of glycosphingolipids of *Sphingomonas* spp and their distribution among members of the alpha-4 subclass of Proteobacteria. J. Ind. Microbiol. Biotechnol..

[19] Kawahara K, Moll H, Knirel YA, Zähringer U (2000). Structural analysis of two glycosphingolipids from the lipopolysaccharide-lacking bacterium *Sphingomonas capsulata*. Eur. J. Biochem..

[20] Kinjo Y (2005). Recognition of bacterial glycosphingolipids by natural killer T cells. Nature.

[21] Mattner J (2005). Exogenous and endogenous glycolipid antigens activate NKT cells during microbial infections. Nature.

[22] Geissmann, F. *et al.* Intravascular immune surveillance by CXCR6+ NKT cells patrolling liver sinusoids. *PLoS Biol*. **3**, e113 (2005). 10.1371/journal.pbio.0030113 PMC1073691 15799695

[23] Kita H (2002). Quantitation and phenotypic analysis of natural killer T cells in primary biliary cirrhosis using a human CD1d tetramer. Gastroenterology.

[24] Tsuneyama K, Yasoshima M, Harada K, Hiramatsu K, Gershwin ME, Nakanuma Y (1998). Increased CD1d expression on small bile duct epithelium and epithelioid granuloma in livers in primary biliary cirrhosis. Hepatology.

[25] Kita H (2002). Analysis of TCR antagonism and molecular mimicry of an HLA-A0201-restricted CTL epitope in primary biliary cirrhosis. Hepatology.

[26] Shimoda S (2000). Mimicry peptides of human PDC-E2 163–176 peptide, the immunodominant T-cell epitope of primary biliary cirrhosis. Hepatology.

[27] Kumagi T, Abe M, Ikeda Y, Hiasa Y (2010). Infection as a risk factor in the pathogenesis of primary biliary cirrhosis: Pros and cons. Dis. Markers..

[28] Bogdanos DP (2004). Microbial mimics are major targets of crossreactivity with human pyruvate dehydrogenase in primary biliary cirrhosis. J. Hepatol..

[29] Harada K, Tsuneyama K, Sudo Y, Masuda S, Nakanuma Y (2001). Molecular identification of bacterial 16S ribosomal RNA gene in liver tissue of primary biliary cirrhosis: Is Propionibacterium acnes involved in granuloma formation?. Hepatology.

[30] Haruta I (2012). Involvement of commensal bacteria may lead to dysregulated inflammatory and autoimmune responses in a mouse model for chronic nonsuppurative destructive cholangitis. J. Clin. Immunol..

[31] Chazouillères O, Wendum D, Serfaty L, Montembault S, Rosmorduc O, Poupon R (1998). Primary biliary cirrhosis-autoimmune hepatitis overlap syndrome: Clinical features and response to therapy. Hepatology.

[32] Caporaso JG (2012). Ultra-high-throughput microbial community analysis on the Illumina HiSeq and MiSeq platforms. ISME J..

[33] Caporaso JG (2010). QIIME allows analysis of high-throughput community sequencing data. Nat. Methods..

[34] Edgar RC (2010). Search and clustering orders of magnitude faster than BLAST. Bioinformatics.

[35] Haas BJ (2011). Chimeric 16S rRNA sequence formation and detection in Sanger and 454-pyrosequenced PCR amplicons. Genome Res..

[36] Lozupone C, Knight R (2005). UniFrac: A new phylogenetic method for comparing microbial communities. Appl. Environ. Microbiol..

[37] Segata N (2011). Metagenomic biomarker discovery and explanation. Genome Biol..

