# Supplementary material for: Ileal mucosa-associated microbiota overgrowth associated with pathogenesis of primary biliary cholangitis
Source: Sci Rep. 2021 Oct 5;11:19705. doi: 10.1038/s41598-021-99314-9 (PMC8492680; doi:10.1038/s41598-021-99314-9)
Supplement: Supplementary file 1 — Supplementary Figure S1. [file 41598_2021_99314_MOESM1_ESM.pdf]

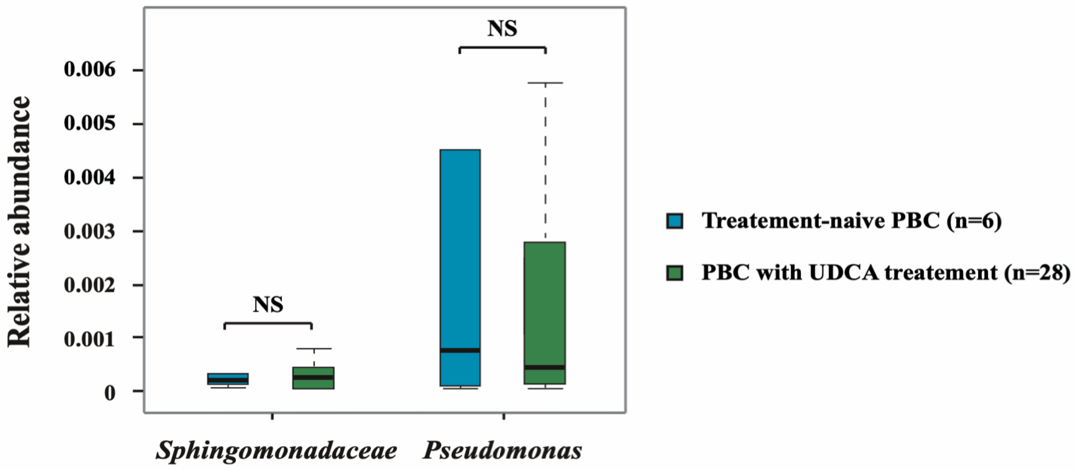

Supplementary figure 1: Relative abundance of *Sphingomonadaceae* and *Pseudomonas* in the treatment-naïve primary biliary cholangitis (PBC) group compared with that in the ursodeoxycholic acid (UDCA)-treated PBC group. The values are expressed as the median of the interquartile range.
