# Supplementary material for: Ileal mucosa-associated microbiota overgrowth associated with pathogenesis of primary biliary cholangitis
Source: Sci Rep. 2021 Oct 5;11:19705. doi: 10.1038/s41598-021-99314-9 (PMC8492680; doi:10.1038/s41598-021-99314-9)
Supplement: Supplementary file 2 — Supplementary Figure S2. [file 41598_2021_99314_MOESM2_ESM.pdf]

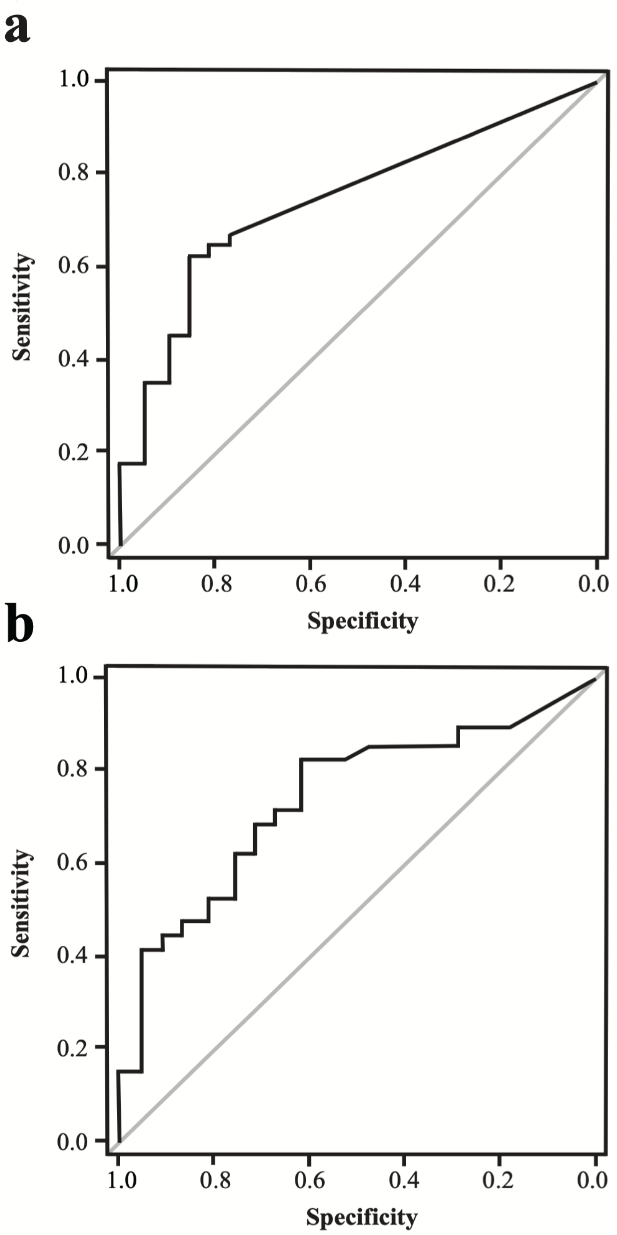

Supplementary figure 2: Characteristics of gut microbiota that distinguishes patients with primary biliary cholangitis (PBC) from healthy individuals

Receiving operational curve analysis was performed for (a) *Sphingomonadaceae* data (PBC: 34; control: 21, AUC = 0.745) and (b) *Pseudomonas* data (PBC: 34; control: 21, AUC = 0.735), respectively. The diagonals indicate a random classification (AUC = 0.5).
