# Supplementary figures and images for: Ileal mucosa-associated microbiota overgrowth associated with pathogenesis of primary biliary cholangitis
Source: Sci Rep. 2021 Oct 5;11:19705. doi: 10.1038/s41598-021-99314-9 (PMC8492680; doi:10.1038/s41598-021-99314-9)

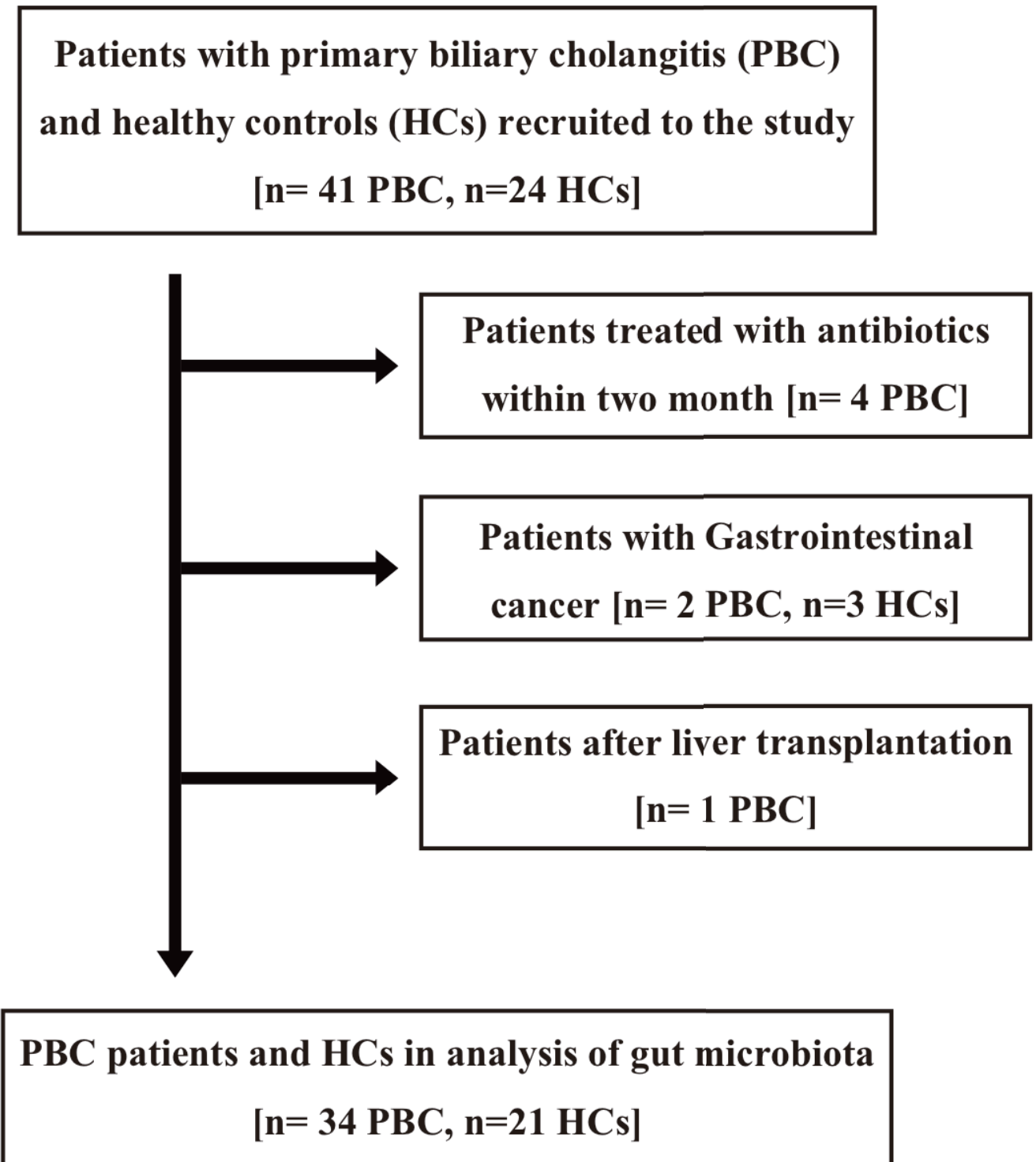

Supplementary figure 3: Patient flow chart of the study

Supplement: Supplementary file 3 — Supplementary Figure S3. [file 41598_2021_99314_MOESM3_ESM.pdf]
