# Supplementary material for: Ileal mucosa-associated microbiota overgrowth associated with pathogenesis of primary biliary cholangitis
Source: Sci Rep. 2021 Oct 5;11:19705. doi: 10.1038/s41598-021-99314-9 (PMC8492680; doi:10.1038/s41598-021-99314-9)
Supplement: Supplementary file 4 — Supplementary Table S1. [file 41598_2021_99314_MOESM4_ESM.docx]

**Supplementary Table 1**

**Ileal mucosa-associated microbiota overgrowth associated with pathogenesis of primary biliary cholangitis**

Shogo Kitahata^1^, Yasunori Yamamoto^2^, Osamu Yoshida^1^, Yoshio Tokumoto^3^, Tomoe Kawamura^2^, Shinya Furukawa^4^, Teru Kumagi^5^, Masashi Hirooka^1^, Eiji Takeshita^6^, Masanori Abe^1^, Yoshiou Ikeda^2^, Yoichi Hiasa^1^

**Supplementary Table 1. Association analysis between PBC and microbiota at the genus level.**

| Family | Genus | Mean relative abundance | | LDA Score |
| --- | --- | --- | --- | --- |
|  |  | PBC (n=34) | HC (n=21) |  |
| *Leptotrichiaceae* | *Leptotrichia* | 2.00E-03 | 0.011 | 3.747 |
| *Unclassified c. TM7_3* | *Unclassified c. TM7_3* | 5.56E-03 | 9.10E-03 | 3.386 |
| *Enterobacteriaceae* | *Morganella* | 0 | 2.03E-04 | 2.133 |
| *Burkholderiaceae* | *Lautropia* | 2.72E-06 | 2.78E-05 | 2.655 |
| *Mogibacteriaceae* | *Mogibacterium* | 2.97E-05 | 4.56E-05 | 2.429 |
| *Coriobacteriaceae* | *Atopobium* | 2.24E-03 | 2.94E-03 | 2.796 |
| *F16* | *Unclassified f. F16* | 9.99E-04 | 2.12E-03 | 2.826 |
| *Erysipelotrichaceae* | *Bulleidia* | 2.79E-03 | 3.14E-03 | 2.706 |
| *Neisseriaceae* | *Eikenella* | 2.20E-05 | 8.26E-05 | 2.104 |
| *Porphyromonadaceae* | *Paludibacter* | 6.21E-05 | 8.60E-05 | 2.110 |
| *Comamonadaceae* | *Curvibacter* | 7.05E-05 | 0 | 2.350 |
| *Moraxellaceae* | *Acinetobacter* | 1.47E-04 | 6.76E-05 | 2.110 |
| *Carnobacteriaceae* | *Carnobacterium* | 8.39E-05 | 1.27E-05 | 2.087 |
| *Sphingomonadaceae* | *Sphingomonas* | 5.22E-04 | 3.81E-05 | 2.468 |
| *Methylobacteriaceae* | *Methylobacterium* | 8.46E-04 | 1.12E-04 | 2.616 |
| *Pseudomonadaceae* | *Pseudomonas* | 7.12E-03 | 7.10E-04 | 3.525 |
| *Clostridiaceae* | *Unclassified f. Clostridiaceae* | 0.045 | 4.97E-03 | 4.237 |
| Bacterial taxa identified as rich in differences between the two groups analyzed by LEfSe (Logarithmic LDA score >2.0).  PBC, primary biliary cholangitis; HC, healthy control; LEfSe, linear discriminant analysis effect size; LDA, linear discriminant analysis | | | | |
