# Supplementary material for: Ileal mucosa-associated microbiota overgrowth associated with pathogenesis of primary biliary cholangitis
Source: Sci Rep. 2021 Oct 5;11:19705. doi: 10.1038/s41598-021-99314-9 (PMC8492680; doi:10.1038/s41598-021-99314-9)
Supplement: Supplementary file 5 — Supplementary Table S2. [file 41598_2021_99314_MOESM5_ESM.docx]

**Supplementary Table 2**

**Ileal mucosa-associated microbiota overgrowth associated with pathogenesis of primary biliary cholangitis**

Shogo Kitahata^1^, Yasunori Yamamoto^2^, Osamu Yoshida^1^, Yoshio Tokumoto^3^, Tomoe Kawamura^2^, Shinya Furukawa^4^, Teru Kumagi^5^, Masashi Hirooka^1^, Eiji Takeshita^6^, Masanori Abe^1^, Yoshiou Ikeda^2^, Yoichi Hiasa^1^

**Supplementary Table 2. The effects of UDCA on PBC-related microbiota.**

|  | *Sphingomonadaceae* rich** rate (%) | Crude OR  (95% CI) | Adjusted OR *  (95% CI) |
| --- | --- | --- | --- |
| UDCA not taking | 3/6 (50) | 1.0 | 1.0 |
| UDCA taking | 18/28 (64.3) | 1.8 (0.304-10.6) | 1.86 (0.272-12.7) |
| *p* value |  | 0.517 | 0.528 |
|  |  |  |  |
|  | *Pseudomonas* rich***  rate (%) | Crude OR  (95% CI) | Adjusted OR *  (95% CI) |
| UDCA not taking | 5/6 (83.3) | 1.0 | 1.0 |
| UDCA taking | 23/28 (82.1) | 0.92 (0.087-9.69) | 1.05 (0.083-13.3) |
| *p* value |  | 0.945 | 0.967 |
|  | | | |
| *Logistic regression test was adjusted for covariates including gender and age. | | | |
| **The cutoff values were 0.0000179. | | | |
| ***The cutoff values were 0.0000684. | | | |
| UDCA, ursodeoxycholic acid; PBC, primary biliary cholangitis; OR, odds ratio; CI, confidence interval. | | | |
